# TAILOR-MS, a Python Package that Deciphers Complex Triacylglycerol Fatty Acyl Structures: Applications for Bovine Milk and Infant Formulas

**DOI:** 10.1021/acs.analchem.0c04373

**Published:** 2021-04-02

**Authors:** Kang-Yu Peng, Malinda Salim, Joseph Pelle, Gisela Ramirez, Ben J. Boyd

**Affiliations:** † Haematology Research Group, The Heart Research Institute, University of Sydney, Newtown, NSW 2042, Australia; ‡ Drug Delivery, Disposition and Dynamics, Monash Institute of Pharmaceutical Sciences, Parkville, VIC 3052, Australia; § Helen Macpherson Smith Trust laboratory at Monash Institute of Pharmaceutical Sciences, Parkville, VIC 3052, Australia; ∥ ARC Centre of Excellence in Convergent Bio-Nano Science and Technology, Monash Institute of Pharmaceutical Sciences, Parkville, VIC 3052, Australia

## Abstract

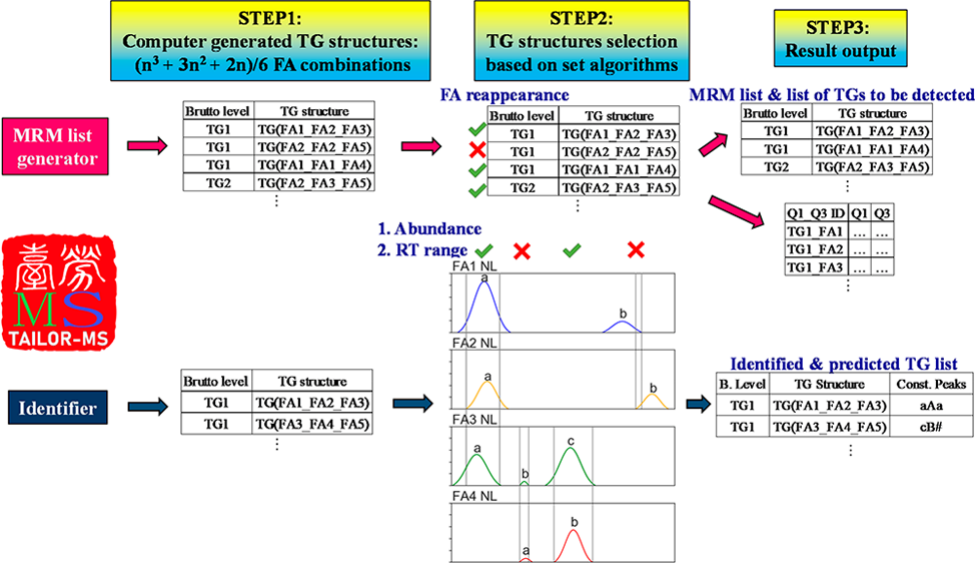

Liquid chromatography tandem mass spectrometry (LC/MS) and other mass spectrometric technologies have been widely applied for triacylglycerol profiling. One challenge for targeted identification of fatty acyl moieties that constitute triacylglycerol species in biological samples is the numerous combinations of 3 fatty acyl groups that can form a triacylglycerol molecule. Manual determination of triacylglycerol structures based on peak intensities and retention time can be highly inefficient and error-prone. To resolve this, we have developed TAILOR-MS, a Python (programming language) package that aims at assisting: (1) the generation of targeted LC/MS methods for triacylglycerol detection and (2) automating triacylglycerol structural determination and prediction. To assess the performance of TAILOR-MS, we conducted LC/MS triacylglycerol profiling of bovine milk and two infant formulas. Our results confirmed dissimilarities between bovine milk and infant formula triacylglycerol composition. Furthermore, we identified 247 triacylglycerol species and predicted the possible existence of another 317 in the bovine milk sample, representing one of the most comprehensive reports on the triacylglycerol composition of bovine milk thus far. Likewise, we presented here a complete infant formula triacylglycerol profile and reported >200 triacylglycerol species. TAILOR-MS dramatically shortened the time required for triacylglycerol structural identification from hours to seconds and performed decent structural predictions in the absence of some triacylglycerol constituent peaks. Taken together, TAILOR-MS is a valuable tool that can greatly save time and improve accuracy for targeted LC/MS triacylglycerol profiling.

## Introduction

Triacylglycerol (TG) is one of the most common lipid classes and possesses several biological functions from being a highly efficient energy storage material to a regulator of cell signaling.^1,2^ TG profiling of biological samples and agricultural and food products has constantly drawn the interest of lipid scientists. Studies regarding TG profiles of various biological samples, such as milk from different mammals and oil from various plant sources, have been widely reported, which often serve important nutritional and quality-control purposes.^3−9^ As a major lipid class, TG is also frequently featured in modern “lipidomics” studies,^4,10,11^

One of the inherent challenges for TG identification is its complex fatty acyl (FA) composition. TG structurally comprises a glycerol backbone and three FA groups. Therefore, *n* number of FAs can theoretically give rise to (*n*^3^ + 3*n*^2^ + 2*n*)/6 TG species (not considering regio- and stereoisomers); that is, up to 220 combinations can be generated from merely 10 FAs.^12^ A widely used approach to identify FA chains constituting TG species is mass spectrometry, which typically involves targeted detection of unique precursor/product ion pairs (MS/MS mode) that are indicative of TG species and their FA groups. Furthermore, chromatographic separation of TG species based on physicochemical properties (e.g., polarity) is often applied prior to mass spectrometric analysis to provide further retention time (RT) information.^13,14^ For example, Li et al. detailed the identification of TG species in soybean seeds by measuring TG ammonium adducts and their multiple neutral losses of FA moieties under positive ion mode with an ESI triple quadrupole mass spectrometer.^6^ Similar approaches have also been implemented to identify bovine milk and infant formula TG composition in numerous other reports using different types of chromatography coupled mass spectrometers.^4,5,8,15−22^ Because one product ion peak only reveals the identity of an FA (with [diacylglycerol]^+^) from the selected TG, matching the detected product ion peaks based on their abundances and RT is necessary in order to decipher TG structures. When conducted manually, this entire process can be laborious, time-consuming, and prone to errors, especially when the sample of interest has a complex TG composition, e.g., bovine milk.^9,15^

Attempts have been made to automate TG structural prediction with input FA information (i.e., species and abundances) using different computational algorithms. However, this type of approach relied heavily on statistical distributions with little biological relevance, and the results were not always accurate.^12,23^ Here, we took an alternative approach and developed TAILOR-MS (acronym for Triacylglycerol Identifier for Low Resolution Mass Spectrometry), a Python package that is capable of automating TG structural identification with input targeted LC/MS data. To evaluate its performance, TAILOR-MS has been applied to characterize TG species in bovine milk and infant formulas. TAILOR-MS exhibited superior TG identification and prediction capabilities compared to manual analysis. With the aid of TAILOR-MS, we generated comprehensive TG profiles of bovine milk and infant formulas containing more detailed composition than most previous reports, which demonstrated the high levels of complexity of the two types of biological samples in terms of TG composition.

## Materials and Methods

### Sample Preparation and ESI-LC-MS/MS TG Detection

Targeted bovine milk and infant formulas TG analysis using ESI-LC-MS/MS was largely similar to our previous work^24^ and was described in detail in the Supporting Information.

### TAILOR-MS

**Figure 1:**
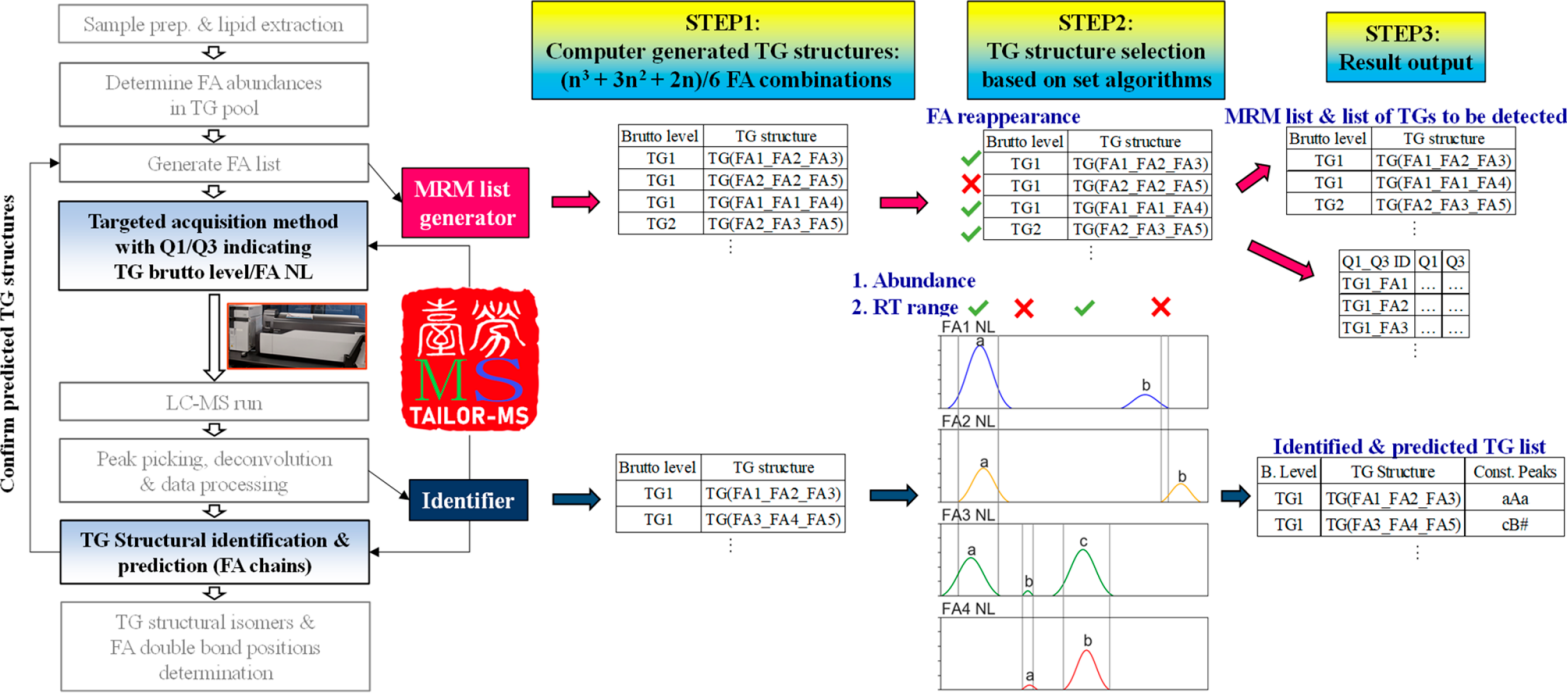
Schematic representations of TAILOR-MS assisted triacylglycerol structure determination. TAILOR-MS aims at expediting LC/MS-based targeted TG species detection and identification process with an extra high accuracy. The package consists of two parts: MRM List Generator and Identifier. The former assists LC-MS acquisition method development, and the latter assists the identification and prediction of TG structures with different FA combinations. The two scripts follow similar general steps: (1) Generate a comprehensive list of (*n*3 + 3*n*2 + 2*n*)/6 TG species based on the combinations of input FA chains. (2) Remove TG species based on selection algorithms; for the MRM List Generator, this is the set “reappearance” rule; for Identifier, peak retention time spans and their relative abundances must meet the set thresholds. (3) Finally, TAILOR-MS MRM List Generator creates an MRM list and a TG structure list which can be used to set up LC/MS acquisition methods for TG detection. For TAILOR-MS Identifier, the exported file is a list containing identified and predicted TG structures from input LC/MS peaks.

TAILOR-MS is designed to automate identifications of three FA chains that constitute TG species using input targeted LC/MS data. The package was written with Python (v.3.6.6) and relied heavily on Pandas (v. 0.25.1) and Numpy (v. 1.17.2). TAILOR-MS consists of two independent scripts, MRM List Generator and Identifier; both were designed following similar concepts (Figure 1).

MRM List Generator creates a comprehensive list of multiple reaction monitoring (MRM) transitions ([M + NH_4_]^+^/[M + NH_4_ – RCOONH_4_]^+^) that cover the combination of TG species based on the FA groups listed by users. It also simultaneously generates a list of TG structures that can be identified with the above MRM transitions.

**Figure 2:**
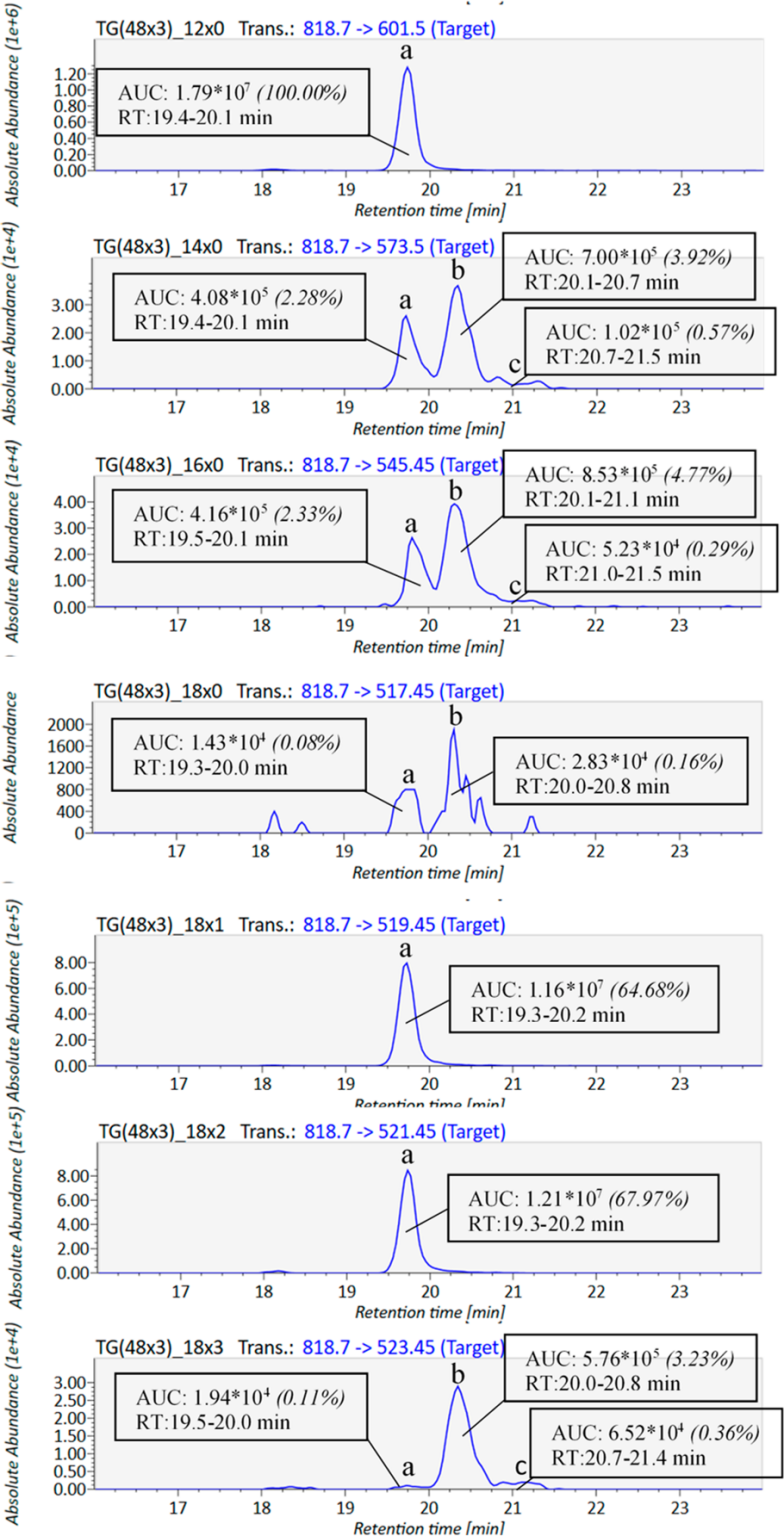
TG structure determination by TAILOR-MS Identifier: an example. The TG structure determination process of infant formula 2 TG(48:3) is presented here to demonstrate how TAILOR-MS works on experimental data. Based on input peak intensity (here AUC), retention time, and peak ID (a, b, and c) information, TAILOR-MS calculates %abundances of the peaks (the most abundant peak as 100%, here TG(48 × 3)_12 × 0), which can be used to exclude low abundance peaks. To confirm the existence of a TG structure, TAILOR-MS checks if a structure is found in a list of *in silico* generated TG structures based on the brutto level and input FA list, as well as peak retention time spans overlap. Furthermore, the script also predicts TG structures based on the difference in TG brutto level and the sum of two in fatty acyl chains that exist in input data (i.e., predicting the third FA chain, # denoted). In this example, TAILOR-MS identifies TG(12:0_18:1_18:2) (aAa), TG(14:0_16:0_18:3) (aaA), TG(14:0_16:0_18:3) (bbB), and TG(14:0_16:0_18:3) (cCc). It also predicts the existence of TG(12:0_14:0_22:3) (aA#), TG(12:0_16:0_20:3) (aA#), TG(14:0_14:0_20:3) (AA#), TG(14:0_14:0_20:3) (BB#), TG(14:0_14:0_20:3) (CC#), and TG(14:0_16:1_18:2) (A#a).

TAILOR-MS Identifier uses processed (peak identification and deconvolution) peak information including names, brutto level (the sum compositional level of identification of TG where the lipid class is followed by the total number of carbons and double bonds across all the constituent FA chains^25,26^), TG (Q1), FA neutral losses (Q3), alphabetic peak IDs which distinguish peaks with the same Q1/Q3 but separated chromatographically, left and right borders of peak RTs, and intensities (can be areas under curves, heights, or concentrations). Two thresholds can be set by the user. The first is % relative abundance, which cuts off TG species that have % relative abundances (vs intensity of the maximal peak among all peaks that share the same Q1, script calculated) below set values. The other is % overlapped retention time, which excludes TG species that do not overlap (based on peak RTs) to the extent above the set values when comparing chromatograms. The rule is the three (or two for prediction) peaks must overlap and the calculated overlapped time segment (of the three or two) must also overlap to or above the set % with the peak of lowest abundance among the three (or two; known as ID peak and indicated by capitalization) that comprise the proposed TG structure. TAILOR-MS Identifier starts by generating a full list of combinations of possible TG structures containing FA information, based on input data. It then identifies TG structures that exist by taking brutto level FA carbon and double bond numbers and then subtracting them with the carbon and double bond numbers of the acquired three or two FA neutral losses. When three neutral losses are present, both numbers post subtraction must be 0. When only two FA neutral losses are provided, TAILOR-MS predicts the third FA and hence full TG structure with the user-defined FA list. The script subsequently drops TG structures for which the three (or two) peaks do not overlap and then excludes TG structures with calculated % relative abundance and % retention time overlap spans below the set thresholds and eventually returns a list of identified and predicted TG species with FA chain information (Figure 1 and Supporting Information Databases 1, 2, and 3). Figure 2 demonstrates how TAILOR-MS determines the structures of TG(48:3) in an infant formula sample. TAILOR-MS executables, example input data, and a manual are provided as Supporting Information files. The source codes can be found on the code repository Web site Github (https://github.com/kangyup/TAILOR-MS).

### Data Analysis and Plotting

Data processing and deconvolution were carried out with MRMPROBS (v. 2.46).^27^ The heatmap and box plots were generated using the Python scientific plotting packages Matplotlib (v. 3.0.3) and Seaborn (v. 0.9.0). Randomized subset selections for assessing the predictive performance of TAILOR-MS Identifier were achieved using Scikit-learn (v. 0.20.0).

## Results

### Use of TAILOR-MS in Bovine Milk and Infant Formula TG Profiling

Bovine milk is known for its complex but well documented TG profile.^10,28,29^ This makes it a suitable sample for evaluating the performance of TAILOR-MS. As a comparison, we also examined TG profiles of two infant formulas.

### TAILOR-MS MRM List Generator

TAILOR-MS is capable of generating a comprehensive MRM list that detects Q1/Q3 ammonium adducts of a TG species and its FA neutral loss fragments, a well-established ESI-LC/MS TG detection method.^13^ Here we first tested this function with 18 most abundant FA groups found in bovine milk and infant formulas TG pools. It is noteworthy that the abundances of these selected FA groups still varied dramatically and that 14:0, 16:0, 16:1, 18:0, 18:1, and 18:2 were among the most abundant (>5%) FA species.^24^ To reduce the number of MRM transitions included in our LC/MS acquisition method and improve mass spectrometric detection quality (by having fewer transitions at a time point), only the above FA groups were allowed to reappear more than once in a TG structure, which could be set with a user defined parameter in input data for the TAILOR-MS MRM List Generator. By doing this, the number of MRM transitions reduced from 1764 to a palatable size of 504, which could determine 308 TG structures without considering regioisomers.

### TAILOR-MS Identifier

**Figure 3:**
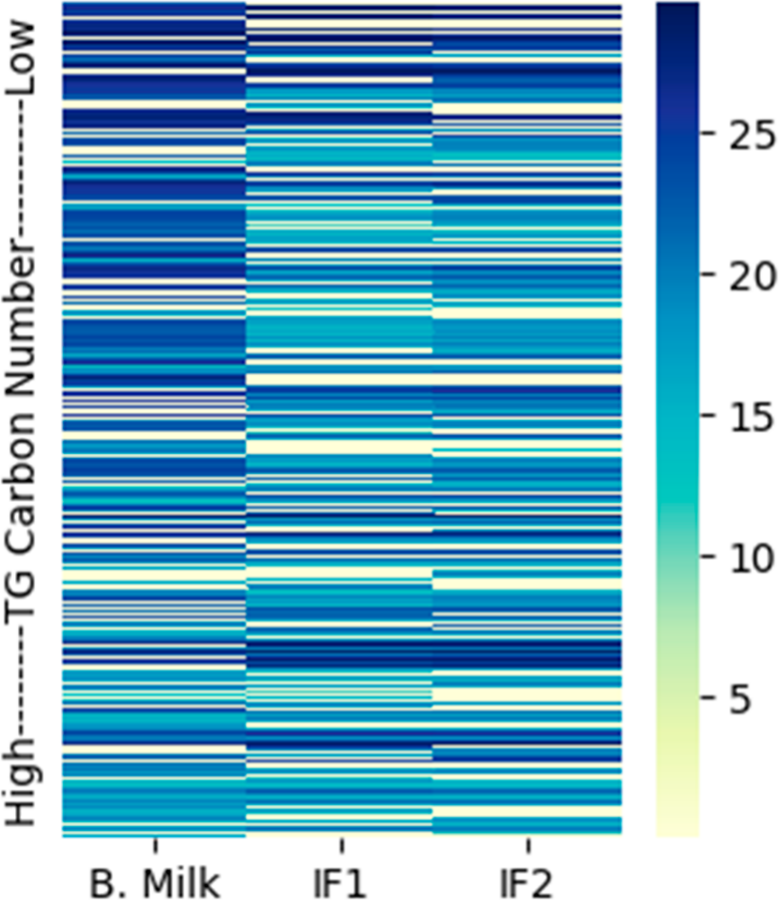
Heatmap presentations of bovine milk and infant formulas TG profiles. The abundances of TAILOR-MS identified TG species in bovine milk and infant formulas are plotted here as heatmaps using log2(AUC) values of the ID peaks. From left panel to right: bovine milk, infant formula 1, infant formula 2. The intensities are indicated by the bar on right. To enable log transformations, TG species with AUC = 0 are substituted by the value 0.1.

**Figure 4:**
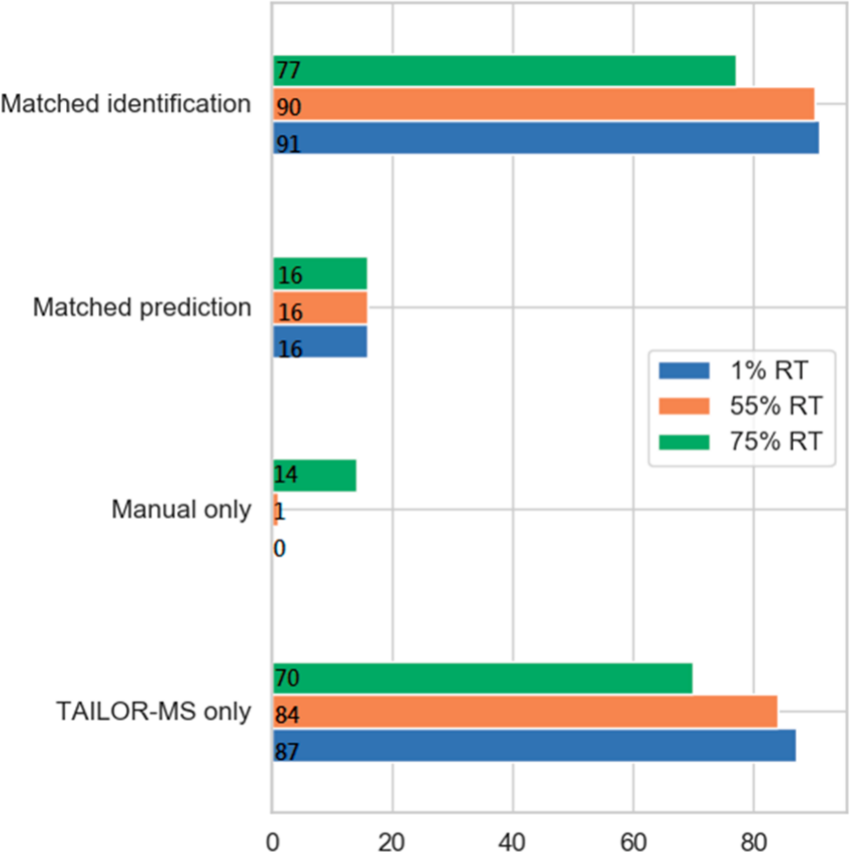
Comparison between manual and TAILOR-MS bovine milk TG identification results. Manually identified TG structures based on a subset (the more abundant TG based on brutto levels) of the data set reported in this study (Supporting Table 2), which was used previously for TG species determination for other studies. The manual result was compared to the TG structures identified by TAILOR-MS. TAILOR-MS successfully identified/predicted all the structures in our previous manual work. It also distinguished structures which should be excluded if adequate retention time span overlap of the three constituent FA neutral loss ion peaks was considered (below the set thresholds; here the results of 1%, 55%, and 75% RT tolerances). TAILOR-MS further identified 70–87 TG structures, depending on the set RT tolerance, that were not manually identified.

To test the second function of TAILOR-MS, we took unpublished bovine milk and infant formula LC/MS data that were initially used to develop LC/MS lipid profiling methods in one of our earlier publications.^24^ These data sets were generated prior to the development of TAILOR-MS. However, similar procedures were followed when we manually created LC/MS acquisition methods. In total, data with 373 different MRM transitions were recorded, which contained information on 544 peaks. With these input data, TAILOR-MS generated 564 possible TG species with FA structural details in bovine milk (settings: 0% relative abundance; 75% retention time span; 33 FA groups in FA list; same for infant formulas below). Among them, 247 identified TG species had all their 3 FA peaks detected by our targeted method, whereas the other 317 TG structures were predicted based on 2 detected FA peaks, with the third FA filled from the input FA list. The numbers of TG species being identified in infant formulas were similar and both fewer than that of bovine milk. In total, 473 TG species were found in infant formula 1; 209 were identified by 3 FA peaks; 264 were predicted based on 2 detected FA peaks. For infant formula 2, the numbers were 486, 206, and 280, respectively. The similarities of these samples were visualized with a heatmap plot (Figure 3). Heatmap patterns indicated that the TG composition of bovine milk was less similar to either of the infant formulas than between the two infant formulas themselves, and TG species with shorter chains were more abundant in bovine milk than in infant formulas (Figure 3). The bovine milk and infant formula TG composition obtained here was also compared to bovine milk TG composition reported earlier,^28^ which showed a greater resemblance to our bovine milk TG profile but was less similar to the infant formulas (Supporting Table 1). Furthermore, when we compared TAILOR-MS generated bovine milk TG structures to manually determined TG structures from our previous work (Supporting Table 2),^24,30^ TAILOR-MS clearly outperformed the manual work, as it not only picked up all the structures we determined by hand but also excluded TG structures with mismatched retention time spans and identified additional structures we missed when performing this task manually (Figure 4).

TG regioisomers (also known as positional isomers) have been reported in bovine milk, and TAILOR-MS is capable of labeling chromatographically separated regioisomers.^15,16^ With our input LC/MS data, a large number of TG regioisomers have been identified or predicted. More positional isomers were identified/predicted in bovine milk than in infant formulas. In fact, more than 50% of the TG species in bovine milk have at least one positional isomer, whereas for infant formula 1 and 2 the percentages were 34.0% and 39.3%, respectively. The finding partially explains why more TG species have been found in bovine milk than infant formulas, despite the similar input MRM transitions (Table 1).

**Table 1:** Numbers of TG Regioisomers^a^

**No. Isomers**	**Bovine Milk**	**Infant** Formula 1	**Infant** Formula 2
**1**	275	312	295
**2**	268	140	158
**3**	21	21	33

a These numbers contain both identified and predicted TG species. The existence of predicted TG species needs to be confirmed. Actual regioisomer numbers can be less (See the Limitations and Caveats section in the Discussion).

### Assessing the Performance of TAILOR-MS Identifier

The use of TAILOR-MS could greatly expedite the TG species identification process, and this has been demonstrated by the calculated average script run time with input bovine milk and infant formula data. As shown in Table 2, the script run time was typically around or less than 30 s, which appeared to be positively related to the size of input data. This was a substantial improvement based on our prior experience in manually identifying these TG species using the same input data, which took several hours to complete.

**Table 2:** Computation Time for TAILOR-MS Identifier^a^

	**Bovine Milk**	**Infant** Formula 1	**Infant** Formula 2
**No. input peaks**	544	498	484
**Avg run time (s)**	30.4 ± 0.7	26.1 ± 0.6	25.9 ± 0.2

a Test runs were performed on an Asus X542U laptop (Taipei, Taiwan). The run time was shown as mean ± sem of 10 separate runs.

**Figure 5:**
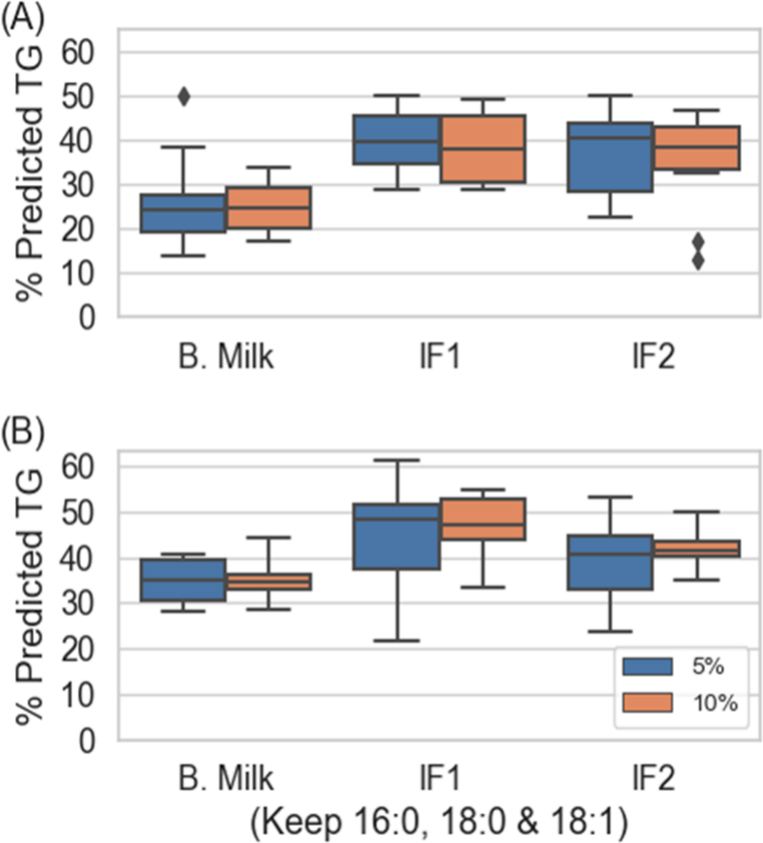
Prediction performances of TAILOR-MS Identifier. Prediction accuracies of TAILOR-MS Identifier were tested here by randomly taking data subsets containing 95% and 90% (equivalent to 5% and 10% missing, as labeled in the figure) of the input peaks from bovine milk (B. Milk), infant formula 1 (IF1), and infant formula 2 (IF2) input peak lists. Identified TG species from these subsets were compared against their corresponding identified TG species generated from the full lists (i.e., no peaks taken away), and those TG species unidentified by subset input data were compared against predicted TG species also generated by the same subset. If found, the predictions were deemed successful. Percentages of successfully predicted TG species from 10 randomly generated subsets for B. Milk, IF1, and IF2 were summarized as box and whisker plots with (A) randomly discarding 5 or 10% total peaks and (B) randomly discarding 5 or 10% total peaks, but not the 16:0, 18:0, and 18:1 FA groups.

The predication function of TAILOR-MS Identifier was likewise examined, using a subset-taking approach conceptually similar to cross validation.^31^ 5% or 10% input peak data were randomly removed from the bovine milk and infant formula data sets. The remaining subsets containing 95% and 90% of the original input peak data were run through TAILOR-MS Identifier, and then the outcomes were compared to those generated from their respective full lists of data. Unsurprisingly, fewer TG species were identified in the subset outcomes. Some of these missing TG species nonetheless were captured by the prediction function of TAILOR-MS. Percentage predicted TG species (vs all missing TG species in subset results) were subsequently calculated. The process was repeated using 10 different randomly generated subsets for each data set, and the results are shown as Figure 5. Overall, TAILOR-MS predicted the existence of approximately 25–40% of all the missing TG species (Figure 5A). Notably, 16:0, 18:0, and 18:1 FAs were the top 3 abundant FA groups in all the samples tested here.^24^ In fact, 16- and 18-carbon FA groups are often the major TG constituents in many biological samples.^32,33^ It is unlikely to miss them when setting up a targeted acquisition method. Therefore, we conducted another test similar to the above, except this time we always retained the 16:0, 18:0, and 18:1 FA groups. By doing so, the prediction performance appeared to improve by approximately 10% for bovine milk and infant formula 1 results, but only resulted in slight improvement to the prediction performance for infant formula 2 (Figure 5B). The effects of manipulating input data settings on the numbers of TG structures being generated by TAILOR-MS Identifier are also shown in Supporting Table 3, which gives us an idea about the usage of these different parameters.

## Discussion

### Importance of the Study

TG structure identification can be a time-consuming and arduous task if carried out manually. Our study has demonstrated that TAILOR-MS could tremendously simplify and expedite this process and minimize human errors. With input LC/MS data, TAILOR-MS successfully generated lists of TG structures that existed in bovine milk, which is known to have very complex TG composition. In comparison, the TG composition of two infant formulas was also determined by TAILOR-MS. Although this work focuses on using these systems for validation purposes, the identification of 247 TG species makes it one of the most complete and detailed single reports of bovine milk TG composition to date, with the previous bovine milk TG studies totaling around 300.^4,10,15,16,28,29,34^ Only one very recent study identified substantially more TG species in bovine milk, in which the authors used a semiautomatic approach for TG identification with the aid of a proprietary lipid database.^9^ TAILOR-MS also has predicted the presence of a large number of novel TG species that possibly constituted the bovine milk TG lipidome, which we believe could be a useful source for identifying new bovine milk TG in the future.

As was the case for bovine milk TG profiling, we have also presented here comprehensive TG profiles for infant formulas. Several earlier infant formula TG composition studies have been examined, and none of them reported more than 100 TG species.^17−22^ Our study, by contrast, identified at least 200 TG species in each infant formula tested, which possibly makes it the most complex infant formula TG profile presented in the literature to date. Both our bovine milk and infant formula TG profiles resemble earlier reports in several aspects, which again confirms the performance of TAILOR-MS.

### Advantages of TAILOR-MS

Our results not only verify the validity of TAILOR-MS but also demonstrate several advantages of the scripts we have developed. First, it is easy to use and can potentially save the user hours when setting up targeted LC/MS acquisition methods and performing TG structural identification with input LC/MS data. It also enables the user to set up % relative abundances, % retention time spans, and the numbers of FA groups to use for prediction. Manipulating these parameters is useful under several circumstances such as when the user is only interested in the more abundant TG species and wants to reduce the complexity of their data sets (increase % relative abundances). Alternatively, when the chromatographical separation of peaks is incomplete, which quite often happens due to structural isomer peaks, the % retention time span may also be a useful parameter to control the TG species returned (increase or decrease % overlapped time spans, see Limitations and Caveats section). Another novel feature is its ability to predict TG species that are likely to exist based on the user-provided FA list, which enables the user to explore TG species that potentially exist even when some FA chains are missing in the acquisition method. Finally, when coupled with chromatographic separation of TG regioisomers, TAILOR-MS can identify and assign different suffixes to them and therefore name them differently, which assists the process of distinguishing between different structural isomers.

### Limitations and Caveats

While TAILOR-MS provides a simple and rapid solution to TG identification in complex biological samples, there are nevertheless some caveats when using this package. First, TAILOR-MS is designed for targeted mass spectrometric analysis; that is, the inclusion of a list that contains FA of interest is required both for setting up an acquisition method and for TG structural determination. Just like any targeted work, TAILOR-MS does not predict structures (even if they may exist in the sample) if the constituent FA chains are completely absent. Hence some prior anticipation of FA species likely to be present in the matrix is required.

When conducting TG structure prediction, TAILOR-MS is inclusive rather than exclusive and this may create some ambiguity that needs to be examined by the user. One example is infant formula TG(16:0_16:0_20:3) (AA#). As the neutral loss of 20:3 FA was not recorded in the acquisition method, structural prediction of this TG species by TAILOR-MS was based solely on 16:0 FA, which is one of the most abundant FA chains found in our samples. While TG(16:0_16:0_20:3) (AA#) is a possible structure, the actual abundance of this TG species is likely to be much lower, as the abundance of 20:3 FA in the TG pool is very low.^24^ The observed high TG(52:3)_16:0 neutral loss signal more likely comes from TG(16:0_18:1_18:2) (aaA), as 18:1 and 18:2 FA neutral loss signals were both detected and of similar levels to 16:0 FA (Supporting Figure 1A and Supporting Database 2 and 3). Rerunning the sample and including the missing neutral losses (here 20:3 FA) may be necessary to confirm the existence and obtain more accurate abundances of TG species predicted only by 2 FA chains. % relative abundance and intensity values generated by TAILOR-MS are based on the ID peak of the identified TG species, which indicate the highest possible abundance and intensity a TG species can have. When an FA chain forms multiple TG structures, abundances of these TG species can only reflect the sum of them, such as the TG mixtures found in TG(36:0) and TG(38:0). In such cases, the user may consider listing all possible TG structures that share the same ID peak in ways similar to previous reports.^15,16^

Good chromatographic separation of peaks is also key to accurate TG structural identification and prediction. Theoretically, if a TG species comprises three FA chains, the neutral loss peaks arising from these three FA moieties should overlap entirely. However, for samples having complex TG composition such as bovine milk, it is difficult to fully separate all peaks even with long run time and a gradient solvent system.^8^ Some possible scenarios include partially merged adjacent peaks (reduced retention time spans of both peaks; Supporting Figure 1B), two or more merged peaks regarded as one peak (possibly increased retention time span; Supporting Figure 1C), and peak tailing due to issues with chromatographic separation (increased retention time span). TAILOR-MS does not consider these situations and matches overlapped peaks only based on input retention time values. To avoid missing these species, the user can take a more inclusive approach by setting up a lower retention time threshold so that fewer TG structures are removed due to unmatched retention time spans.

Finally, it is noteworthy that TG *sn* positions are not determined by TAILOR-MS. Although TAILOR-MS can distinguish and label TG regioisomers that have been chromatographically separated, it does not suggest positions of the 3 FA chains on the TG glycerol backbone. Nor does it indicate branched and double bond positions on FA chains. Several enzymatic, chromatographic, and mass spectrometric solutions may be implemented to obtain more detailed isomeric TG information.^13,14,35,36^

## Conclusions

TAILOR-MS is a novel Python-based package aiming at automating the FA chain identification and prediction tasks for TG profiling with input LC/MS (or mass spectrometry in general) data. It provides a simple, efficient and accurate solution to a time-consuming and arduous task, which works particularly well on biological and food samples with complex TG composition. By applying TAILOR-MS, we are able to present some of the most comprehensive TG profiles of bovine milk and infant formulas, which further confirms the capability and reliability of this package. We believe the introduction of TAILOR-MS will tremendously expedite the progress of TG profiling in various biological samples with complex TG composition in the future.

## Supporting Information Available

Supporting methods, figures and tables—detailed bovine milk and infant formulas lipid extraction and targeted LC/MS methods; comparison between TAILOR-MS determined bovine milk and infant formulas TG profiles and an earlier bovine milk TG profiling study; manually identified TG species in bovine milk; impacts of altered input settings on TAILOR-MS performance; example ion peak chromatograms to demonstrate key points when using TAILOR-MS Identifier (PDF)
Supporting database—complete lists of identified and predicted TG species in bovine milk and infant formulas (XLSX)
TAILOR-MS executable files (ZIP)
