# Supplementary material for: TAILOR-MS, a Python Package that Deciphers Complex Triacylglycerol Fatty Acyl Structures: Applications for Bovine Milk and Infant Formulas
Source: Anal Chem. 2021 Apr 2;93(14):5684–90. doi: 10.1021/acs.analchem.0c04373 (PMC8047770; doi:10.1021/acs.analchem.0c04373)
Supplement: Supplementary file 1 — ac0c04373_si_001.pdf [file ac0c04373_si_001.pdf]

Supporting Information

TAILOR-MS, a Python Package that Deciphers Complex Triacylglycerol Fatty Acyl Structures: Applications for Bovine Milk and Infant Formulas

Kang-Yu Peng[1]*, Malinda Salim[2], Joseph Pelle[3], Gisela Ramirez[2], Ben J. Boyd[2,4]*

[1]Haematology Research Group, The Heart Research Institute, University of Sydney, Newtown, NSW, 2042, Australia;

[2]Drug Delivery, Disposition and Dynamics, Monash Institute of Pharmaceutical Sciences, Parkville, VIC, 3052, Australia;

[3]Helen Macpherson Smith Trust laboratory at Monash Institute of Pharmaceutical Sciences, Parkville, VIC, 3052, Australia;

[4]ARC Centre of Excellence in Convergent Bio-Nano Science and Technology, Monash Institute of Pharmaceutical Scienc-es, Parkville, VIC, 3052, Australia;

*Correspondence: Kang-Yu Peng – kang-yu.peng@hri.org.au; Ben J. Boyd − ben.boyd@monash.edu

Table of Contents

S. Materials and Methods S2

Materials S2

Lipid extraction from bovine milk and infant formulas S2

LC/MS data acquisition and processing S2

S. Table 1. List of TG species with >1 % abundances in bovine milk and infant formulas: comparison to bovine milk TG composition in an earlier report S4

S. Table 2. Manually identified TG structures in bovine milk S6

S. Table 3. Impacts of altered input settings on TAILOR-MS identifier performance and numbers of generated TG species S7

S. Figure 1. Examples for TAILOR-MS assisted TG structure identification that may require user's attention S9

S. Table 4. Summary %C.V. for triplicated bovine milk and infant formula peak areas S10

Supporting Materials and Methods

Materials. Commercially available full cream bovine milk was purchased from a local supermarket (Coles, Mount Waverley, VIC, Australia); infant formulas were kindly provided by Medicines for Malaria Venture (Geneva, Switzerland); acetonitrile, ammonium acetate, chloroform and methanol were purchased from Merck Pty. Ltd. (Kilsyth VIC, AU); 1-butanol, 2-propanol and lipid standards: triglyceride mixtures were obtained from Sigma-Aldrich (St. Louis, MO, USA). Triheneicosanoin was from Nu-Chek Prep (Elysian, MN, USA). All the solvents and the salt used for LC/MS experiments were at least of HPLC level.

Lipid extraction from bovine milk and infant formulas. A single-phase lipid extraction method was used to extract TG from bovine milk and two infant formulas of interest, similar to the method reported previously (1). Briefly, 20 μL of bovine milk (containing 3.8 % total lipid) or infant formulas (prepared to contain equivalent amount of total lipid to bovine milk) were taken and mixed with 400 μL of $CHCl_3$:MeOH = 2:1 with a positive displacement pipette. The samples were subsequently shaken for 10 min and sonicated in a water bath for 30 min under room temperature. Afterwards, the samples were centrifuged at 16000g for 10 min. Supernatants were transferred to a 96-well plate and then blown dry by N_2 gas. The sample plate was stored at -20°C and were reconstituted prior to LC/MS analysis. Reconstitution was achieved by adding 50 μL butanol and 50 μL methanol into plate wells containing the samples. The plate was then centrifuged at 2250g for 5 min. The reconstituted samples were transferred to glass vials with inserts for analysis. The experiments were done in triplicate of which 75% triplicate peaks areas have <17.1% C.V. (Supporting Table 4). Triplicate log2 peak areas were presented in Figure 3. All the other results presented in this study were based on peak areas from one of the three replicates.

LC/MS data acquisition and processing. A Shimadzu NexeraX2 UHPLC system tandem Shimadzu #8050 triple quadrupole mass spectrometer system equipped with an ESI module was used for targeted TG data acquisition (Shimadzu Australia, Sydney, NSW). Briefly, 5 μL

of samples were injected to a Waters Symmetry C18 column (100Å, 3.5 µm, spherical Silica, 4.6x75 mm) for chromatographic separation, with 40°C column oven temperature. A gradient was established over 40 minute run time with the following binary solvent system: solvent A: H2O: acetonitrile = 4:6 (v/v); solvent B: acetonitrile: isopropanol =1:9 (v/v), both containing 10 mM ammonium acetate, similar to an earlier report (2)*. Flow rate was set at 0.4 mL/min. The solvent gradient was 40% to 70% B, 0-3 min; 70% to 100% B, 3-35 min; 100% B, 35-37 min; 0% B, 37-40 min. Targeted TG and FA neutral losses detection was carried out with MRM mode, with collision energy set as -11eV. The precursor/product ion pair (Q1/Q3) detected was $[M+NH_4]^+/[M + NH_4–RCOOHNH_3]^+$, which was based on the Lipid Maps database. Commercially available triacylglycerol lipid standards were used to further confirm the retention time and identities of detected triacylglycerol ion peaks. In total, 372 MRM transitions have been included in the acquisition method. A summary of peak area under curve (AUC) %C.V. of the triplicate are presented as Supporting Table 4.

*Prolonged runs (>10 hr) with this solvent system is not recommended (23).

Supporting Table 1. List of TG species with >1 % abundances in bovine milk and infant formulas: comparison to bovine milk TG composition in an earlier report

Bovine Milk (Lit.)*	Bovine Milk*^
TG(4:0_16:0_16:0) [3.20]	**TG(4:0_16:0_16:0)(#CC) [5.55]**
TG(16:0_18:1_18:1) [2.50]	**TG(6:0_16:0_16:0)(#BB) [4.30]**
TG(10:0_16:0_18:1) [1.60]	**TG(4:0_16:0_18:0)(#Cc) / TG(6:0_16:0_16:0)(#CC) [3.56]**
TG(6:0_16:0_16:0) [1.50]	**TG(4:0_16:0_16:0)(#BB) [3.04]**
TG(4:0_16:0_18:1) [4.17]	**TG(16:0_16:0_18:1)(aaA) [3.03]**
TG(4:0_14:0_16:0) [3.05]	**TG(6:0_14:0_16:0)(#Bb) [2.74]**
TG(14:0_16:0_18:1) [2.82]	**TG(6:0_16:0_16:0)(#AA) [2.66]**
TG(4:0_16:0_18:0) [2.47]	TG(10:0_10:0_22:0)(AA#) / TG(10:0_16:0_16:0)(Aaa) [2.54]
TG(16:0_16:0_18:1) [2.34]	TG(4:0_18:0_18:0)(#BB) [2.52]
TG(16:0_18:0_18:1) [2.16]	**TG(6:0_16:0_18:0)(#Bb) [2.36]**
TG(6:0_16:0_18:1) [2.02]	TG(8:0_16:0_16:0)(Aaa) [2.19]
TG(4:0_14:0_18:1) [1.79]	TG(10:0_10:0_20:0)(AA#) / TG(10:0_14:0_16:0)(Aaa) [2.04]
TG(4:0_18:0_18:1) [1.58]	TG(6:0_18:0_18:0)(#AA) [2.01]
TG(4:0_18:1_18:1) [1.48]	TG(14:0_14:0_20:1)(BB#) / **TG(14:0_16:0_18:1)(Bbb) [1.94]**
TG(14:0_18:0_18:1) [1.45]	**TG(10:0_16:0_18:1)(Aaa) [1.85]**
TG(6:0_14:0_16:0) [1.37]	TG(10:0_14:0_18:0)(aAa) / TG(14:0_14:0_14:0)(AAA) [1.59]
TG(4:0_14:0_18:0) [1.35]	TG(16:0_16:0_20:2)(AA#) / **TG(16:0_18:1_18:1)(Aaa) [1.54]**
TG(14:0_18:1_18:1) [1.26]	TG(14:0_14:0_16:0)(BBb) [1.43]
TG(12:0_16:0_18:1) [1.22]	**TG(6:0_14:0_16:0)(#Cc) [1.36]**
TG(18:0_18:1_18:1) [1.21]	TG(8:0_8:0_22:0)(AA#) [1.31]
TG(6:0_16:0_18:0) [1.12]	TG(12:0_14:0_18:0)(Bbb) / TG(12:0_16:0_16:0)(Bbb) [1.30]
TG(18:1_18:1_18:1) [1.02]	TG(10:0_14:0_14:0)(Aaa) [1.27]
	**TG(18:1_18:1_18:1)(AAA) [1.26]**
	TG(14:0_14:0_18:1)(AAa) [1.23]
	TG(12:0_12:0_22:1)(AA#) / TG(12:0_14:0_20:1)(Aa#) / **TG(12:0_16:0_18:1)(Aaa) [1.14]**
	**TG(4:0_16:0_18:0)(#bB) [1.09]**
	TG(10:0_10:0_22:1)(AA#) [1.08]
	TG(8:0_16:0_18:1)(aBb) [1.06]
	TG(16:0_16:0_20:1)(AA#) / **TG(16:0_18:0_18:1)(Aaa) [1.05]**
	TG(14:0_16:0_16:0)(Bbb) [1.04]
	TG(10:0_12:0_20:0)(aA#) / TG(12:0_12:0_18:0)(AAa) / TG(12:0_14:0_16:0)(Aaa) [1.03]
	TG(8:0_14:0_20:1)(aA#) [1.03]

Infant Formula 1*^	Infant Formula 2*^
TG(12:0_12:0_12:0)(AAA) [6.63]	TG(12:0_12:0_12:0)(AAA) [7.55]
**TG(18:1_18:1_18:1)(AAA) [5.15]**	TG(16:0_16:0_20:2)(AA#) [6.03]
TG(16:0_16:0_20:2)(AA#) [5.06]	**TG(16:0_18:1_18:1)(aAA) [5.99]**
**TG(16:0_18:1_18:1)(aAA) [5.0]**	**TG(18:1_18:1_18:1)(AAA) [5.56]**
TG(12:0_12:0_14:0)(aaA) [4.87]	TG(12:0_12:0_14:0)(aaA) [5.36]
**TG(16:0_16:0_18:1)(aaA) [4.19]**	**TG(16:0_16:0_18:1)(aaA) [4.46]**
TG(12:0_14:0_14:0)(aAA) [3.93]	TG(16:0_16:0_20:3)(AA#) [4.13]
TG(12:0_12:0_16:0)(aaA) [3.68]	TG(12:0_14:0_14:0)(aAA) [3.98]
TG(8:0_8:0_18:0)(AA#) / TG(8:0_12:0_14:0)(Aa#) [3.29]	TG(12:0_12:0_16:0)(aaA) [3.77]
TG(16:0_16:0_20:3)(AA#) [3.23]	TG(16:0_18:1_18:2)(aaA) [3.49]
TG(8:0_10:0_16:0)(aA#) / TG(10:0_10:0_14:0)(AA#) / TG(10:0_12:0_12:0)(Aaa) [3.08]	TG(16:0_16:0_20:1)(AA#) [3.04]

TG(10:0_12:0_14:0)(Aaa) [3.07]	TG(10:0_10:0_14:0)(AA#) / TG(10:0_12:0_12:0)(Aaa) [2.97]
TG(16:0_16:0_20:1)(AA#) [3.01]	TG(8:0_8:0_16:0)(AA#) / TG(8:0_12:0_12:0)(Aaa) [2.58]
TG(8:0_8:0_16:0)(AA#) / TG(8:0_12:0_12:0)(Aaa) [2.99]	TG(16:0_16:0_18:2)(aaA) [2.35]
**TG(4:0_16:0_16:0)(#AA) / TG(6:0_14:0_16:0)(#aA) / TG(10:0_10:0_16:0)(aaA) [2.76]**	TG(12:0_12:0_18:1)(aaA) [2.28]
TG(8:0_8:0_20:0)(AA#) / TG(8:0_10:0_18:0)(Aa#) / TG(8:0_12:0_16:0)(Aaa) / TG(8:0_14:0_14:0)(Aaa) [2.69]	TG(8:0_8:0_18:0)(AA#) / TG(8:0_10:0_16:0)(Aa#) / TG(8:0_12:0_14:0)(Aa#) [2.26]
TG(16:0_18:1_18:2)(aaA) [2.63]	**TG(16:0_18:0_18:1)(aaA) [2.15]**
TG(16:0_16:0_18:2)(aaA) [2.38]	TG(18:1_18:1_18:2)(aaA) [2.15]
**TG(6:0_16:0_16:0)(#AA) [2.32]**	TG(10:0_12:0_14:0)(Aaa) [2.11]
TG(18:1_18:1_18:2)(aaA) [2.25]	**TG(18:0_18:1_18:1)(Aaa) [2.02]**
TG(14:0_14:0_14:0)(AAA) [2.06]	TG(12:0_14:0_16:0)(aAa) / TG(14:0_14:0_14:0)(AAA) [1.34]
**TG(16:0_18:0_18:1)(aaA) [2.06]**	TG(16:0_16:0_16:0)(AAA) [1.19]
TG(12:0_14:0_16:0)(Aaa) [2.01]	TG(8:0_8:0_20:0)(AA#) / TG(8:0_10:0_18:0)(Aa#) / TG(8:0_14:0_14:0)(Aaa) [1.17]
**TG(18:0_18:1_18:1)(Aaa) [1.91]**	TG(12:0_12:0_20:1)(AA#) [1.11]
TG(10:0_12:0_16:0)(Aaa) / TG(10:0_14:0_14:0)(Aaa) [1.69]	TG(12:0_14:0_18:1)(aAa) [1.01]
TG(6:0_18:0_18:0)(#AA) / TG(12:0_12:0_18:0)(aaA) [1.17]	**TG(6:0_16:0_16:0)(#AA) [1.00]**
TG(8:0_8:0_22:0)(AA#) / TG(8:0_12:0_18:0)(Aaa) / TG(8:0_14:0_16:0)(Aaa) [1.01]	
**TG(4:0_16:0_18:0)(#aA) / TG(6:0_14:0_18:0)(#aA) [1.01]**	

*: The number in [] is % of the TG species. For bovine milk reported in the literature, this is mol %; for bovine milk and infant formulas tested in our study, it is % of summed AUC, which corresponds to mol %. Bovine milk TG composition was obtained from reference (27).

^ : Contents in the brackets after the TG species represent the neutral loss peaks used to identify this TG species. When more than one TG species share an ID peak, all the possible TG species are listed. See TAILOR-MS method section and the manual for TAILOR-MS usage for detailed explanations.

Supporting Table 2. Manually identified TG structures in bovine milk.

Brutto	S1	S2	S3	S4	S5	S6
TG(38:0)	TG(12:0_12:0_14:0)	TG(10:0_14:0_14:0)	TG(6:0_14:0_18:0)	TG(10:0_12:0_16:0)	TG(6:0_16:0_16:0)	TG(4:0_16:0_18:0)
TG(36:0)	TG(12:0_12:0_12:0)	TG(10:0_12:0_14:0)	TG(8:0_12:0_16:0)	TG(6:0_14:0_16:0)	TG(4:0_16:0_16:0)	
TG(40:0)	TG(12:0_12:0_16:0)	TG(8:0_16:0_16:0)	TG(6:0_16:0_18:0)	TG(12:0_14:0_14:0)		
TG(38:1)	TG(8:0_12:0_18:1)	TG(10:0_16:0_16:0)				
TG(42:0)	TG(12:0_14:0_16:0)	TG(8:0_16:0_18:0)	TG(12:0_12:0_18:0)	TG(10:0_14:0_18:0)		
TG(42:1)	TG(12:0_12:0_18:1)	TG(8:0_16:0_18:1)	TG(10:0_14:0_18:1)			
TG(50:1)	TG(16:0_16:0_18:1)	TG(14:0_18:0_18:1)				
TG(52:2)	TG(16:0_18:1_18:1)	TG(16:0_18:0_18:2)				
TG(44:1)	TG(12:0_14:0_18:1)	TG(10:0_16:0_18:1)	TG(8:0_18:0_18:1)			
TG(44:0)	TG(12:0_16:0_16:0)	TG(14:0_14:0_16:0)	TG(12:0_14:0_18:0)	TG(10:0_16:0_18:0)		
TG(48:1)	TG(14:0_16:0_18:1)	TG(14:0_16:1_18:0)	TG(12:0_16:0_16:1)	TG(12:0_18:0_18:1)		
TG(46:1)	TG(12:0_16:0_18:1)	TG(14:0_14:0_18:1)	TG(14:0_14:1_18:0)	TG(14:1_16:0_16:0)		
TG(50:2)	TG(16:0_16:0_18:2)	TG(14:0_18:1_18:1)	TG(16:0_16:1_18:1)			
TG(46:0)	TG(14:0_16:0_16:0)	TG(14:0_14:0_18:0)	TG(12:0_16:0_18:0)			
TG(52:1)	TG(16:0_18:0_18:1)					
TG(34:0)	TG(10:0_12:0_12:0)	TG(8:0_12:0_14:0)				
TG(32:0)	TG(8:0_12:0_12:0)					
TG(48:0)	TG(16:0_16:0_16:0)	TG(14:0_16:0_18:0)	TG(12:0_18:0_18:0)			
TG(48:2)	TG(12:0_18:1_18:1)	TG(14:0_16:0_18:2)	TG(14:1_16:0_18:1)			
TG(54:3)	TG(18:1_18:1_18:1)	TG(18:0_18:1_18:2)				
TG(46:2)	TG(10:0_18:1_18:1)	TG(12:0_16:0_18:2)	TG(14:0_14:1_18:1)	TG(14:0_14:0_18:2)		
TG(44:2)	TG(8:0_18:1_18:1)	TG(12:0_14:0_18:2)				
TG(54:2)	TG(18:0_18:1_18:1)					
TG(40:1)	TG(8:0_14:0_18:1)	TG(10:0_12:0_18:1)	TG(12:0_12:0_16:1)			
TG(50:0)	TG(16:0_16:0_18:0)					
TG(52:3)	TG(16:0_18:1_18:2)	TG(16:1_18:1_18:1)	TG(16:1_18:0_18:2)			
TG(49:1)	TG(15:0_16:0_18:1)	TG(16:0_16:0_17:1)	TG(14:0_17:0_18:1)			
TG(43:0)	TG(12:0_15:0_16:0)	TG(14:0_14:0_15:0)	TG(12:0_14:0_17:0)			
TG(47:0)	TG(15:0_16:0_16:0)	TG(14:0_15:0_18:0)	TG(14:0_16:0_17:0)			
TG(51:1)	TG(16:0_17:0_18:1)	TG(15:0_18:0_18:1)				
TG(51:2)	TG(15:0_18:1_18:1)	TG(16:0_17:1_18:1)				
TG(45:0)	TG(14:0_15:0_16:0)	TG(12:0_15:0_18:0)	TG(12:0_16:0_17:0)			
TG(54:1)	TG(16:0_18:1_20:0)	TG(18:0_18:0_18:1)				
TG(54:4)	TG(18:1_18:1_18:2)					
TG(52:4)	TG(16:0_16:0_20:4)	TG(16:0_18:1_18:3)				
TG(54:5)	TG(18:1_18:1_18:3)	TG(16:0_18:1_20:4)				
TG(47:1)	TG(14:0_15:0_18:1)	TG(15:0_16:0_16:1)				
TG(50:3)	TG(16:0_16:0_18:3)	TG(14:1_18:1_18:1)	TG(14:0_18:1_18:2)			
TG(52:0)	TG(16:0_18:0_18:0)	TG(16:0_16:0_20:0)				
TG(49:0)	TG(15:0_16:0_18:0)	TG(16:0_16:0_17:0)				

The dataset used is a sub-dataset (with only the more abundant brutto level TG) of the input bovine milk TG dataset used for TAILOR-MS automated TG structure identification. A total of 107 TG species have been identified manually.

Supporting Table 3. Impacts of altered input settings on TAILOR-MS identifier performance and numbers of generated TG species.

		Bovine Milk				Infant Formula 1				Infant Formula 2			
		**Total[@]**	**I[@]**	**P[@]**	**Run Time (sec.)**	**Total***	**I[@]**	**P[@]**	**Run Time (sec.)**	**Total***	**I[@]**	**P[@]**	**Run Time (sec.)**
% Relative Abundance	**0**	564	247	317	29.24	473	209	264	24.18	486	206	280	24.57
	**1**	497	215	282	28.99	348	165	183	24.21	371	161	210	24.53
	**5**	415	177	238	29.17	249	120	129	24.15	263	121	142	24.43
	**10**	359	150	209	29.12	208	101	107	24.13	215	102	113	24.42
	**20**	241	97	144	28.93	162	78	84	24.10	171	78	93	24.46
	**% diff**												
	**1vs0**	-11.88	-12.96	-11.04		-26.43	-21.05	-30.68		-23.66	-21.84	-25.00	
	**5vs0**	-26.42	-28.34	-24.92		-47.36	-42.58	-51.14		-45.88	-41.26	-49.29	
	**10vs0**	-36.35	-39.27	-34.07		-56.03	-51.67	-59.47		-55.76	-50.49	-59.64	
	**20vs0**	-57.27	-60.73	-54.57		-65.75	-62.68	-68.18		-64.81	-62.14	-66.79	
% Retention Time	**55**	645	299	346	29.09	570	257	313	24.24	578	260	318	24.63
	**65**	614	277	337	29.07	525	235	290	24.24	528	230	298	24.46
	**75**	564	247	317	29.24	473	209	264	24.18	486	206	280	24.57
	**85**	465	198	267	29.01	412	168	244	24.23	427	171	256	24.60
	**95**	330	121	209	29.07	324	113	211	24.24	314	94	220	24.56
	**% diff**												
	**65vs55**	-4.81	-7.36	-2.60		-7.89	-8.56	-7.35		-8.65	-11.54	-6.29	
	**75vs55**	-12.56	-17.39	-8.38		-17.02	-18.68	-15.65		-15.92	-20.77	-11.95	
	**85vs55**	-27.91	-33.78	-22.83		-27.72	-34.63	-22.04		-26.12	-34.23	-19.50	
	**95vs55**	-48.84	-59.53	-39.60		-43.16	-56.03	-32.59		-45.67	-63.85	-30.82	

FA Number^	**33**	564	247	317	29.24	473	209	264	24.18	486	206	280	24.57
	**20**	402	247	155	23.53	337	209	128	19.01	337	206	131	19.09
	**10**	293	237	56	20.87	247	201	46	16.51	247	200	47	16.34
	**5**	248	228	20	17.14	217	194	23	13.74	214	194	20	15.00
	**% diff**												
	**20vs33**	-28.72	0.00	-51.10		-28.75	0.00	-51.52		-30.66	0.00	-53.21	
	**10vs33**	-48.05	-4.05	-82.33		-47.78	-3.83	-82.58		-49.18	-2.91	-83.21	
	**5vs33**	-56.03	-7.69	-93.69		-54.12	-7.18	-91.29		-55.97	-5.83	-92.86	

@: Total, "I" and "P" columns indicate the number of TG species generated by TAILOR-MS identifier. "I (identified)" means all 3 FAs constituting the structure of a TG species are based on FA neutral losses in the acquisition method, whereas "P (predicted)" means the third FA is predicted by TAILOR-MS based on the given FA list. Total is the sum of "I" and "P" FAs.

^: The FA groups used here are based on their abundances, with the FAs of lower abundances being removed first i.e., the FA list of 5 contains the 5 most of abundant FA groups determined by the profiles of digested bovine milk and infant formulas.

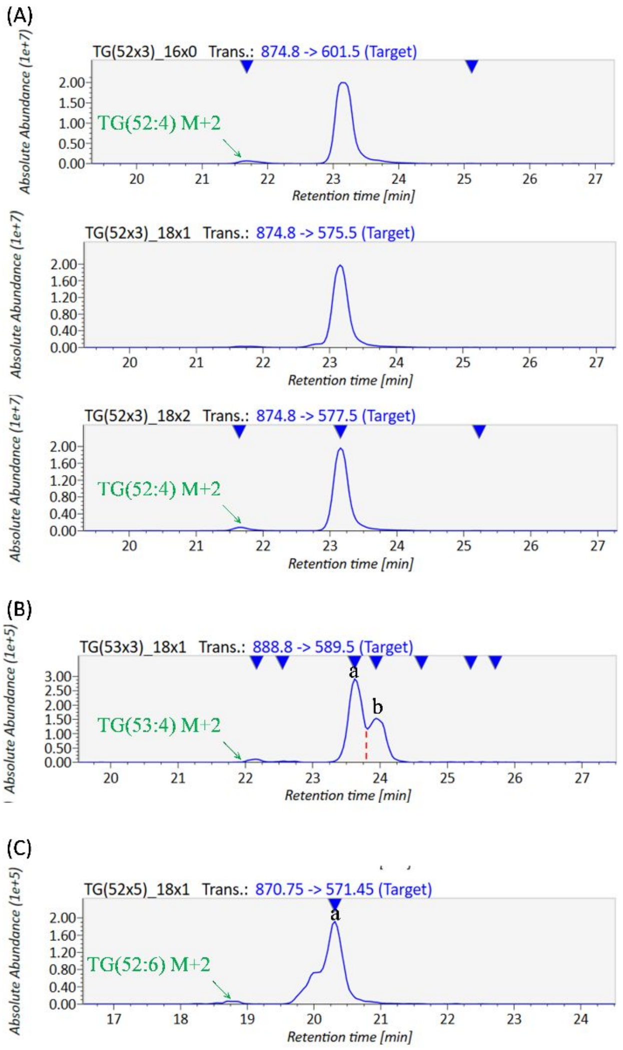

Supporting Figure 1. Examples for TAILOR-MS assisted TG structure identification that may require user's attention. Several infant formula TG ion peak examples are shown here to demonstrate key data interpretation and processing points. As panel A shows, TAILOR-MS may predict a structure that is less likely to exist or with very low abundance, especially when the prediction is based solely on one known fatty acyl moiety. While TG(16:0_16:0_20:3) (AA#) is a predicted structure, it is more likely that most of the fatty acyl 16:0 neutral loss was contributed by the fragmentation of TG(16:0_18:1_18:2), as 18:1 and 18:2 neutral losses both have similar high abundance to 16:0. Also, the abundance of 20:3 in TG is expected to be low. Measuring the neural loss of 20:3 from TG(52:3) will be necessary to confirm the existence of TG(16:0_16:0_20:3). If the two peaks are separated enough to be distinguished (e.g., peak a and b in panel B), the expected peak elution time spans for both peaks are smaller than similar standalone peaks, as parts of the two peaks merge. A smaller retention time tolerance may be set to avoid missing TG structures containing these fatty acyl ion peaks when performing TG structure determination using TAILOR-MS. Alternatively, a partial separation can occur with only one discernible apex (e.g., peak a in panel C). In this case, the user must decide if they view this as one or two peaks and integrate the peak(s) accordingly. If viewed as one peak, this peak will have a larger retention time span.

Supporting Table 4. Summary of triplicate bovine milk and infant formula peak area %C.V.

% C.V.	B. Milk	IF1	IF2
**count**	543	498	484
**mean**	11.2	10.9	13.2
**std**	14.0	10.1	9.3
**min**	0.4	0.6	0.6
**25%**	3.7	5.3	7.0
**median**	6.8	8.3	11.8
**75%**	11.8	12.7	17.1
**max**	88.8	125.2	85.1
