# Supplementary material for: TAILOR-MS, a Python Package that Deciphers Complex Triacylglycerol Fatty Acyl Structures: Applications for Bovine Milk and Infant Formulas
Source: Anal Chem. 2021 Apr 2;93(14):5684–90. doi: 10.1021/acs.analchem.0c04373 (PMC8047770; doi:10.1021/acs.analchem.0c04373)
Supplement: Supplementary file 3 — ac0c04373_si_003.zip [file ac0c04373_si_003.zip › TAILOR-MS/TAILOR-MS_User_Manual.docx]

**TAILOR-MS**

**TAILOR-MS** (Triacylglycerol Identifier for Low Resolution Mass Spectrometry) is a Python package for automating the triacylglycerol identification process with input targeted LC-MS (or other types of mass spectrometric data) data. It consists of two independent scripts: **MRM List Generator** and **Identifier**.

**MRM List Generator**

This script creates an MRM list for setting up targeted mass spectrometric acquisition methods for TG with [M+NH_4_]^+^/[ M+NH_4_-RCOONH_4_]^+^ precursor/product ion pairs. After unzipping TAILOR-MS_MRM_generatior.zip file, you will find in folder “**FA_MRM.csv**” and “**TAILOR-MS_MRM_generator.exe**” files. To use MRM generator, simply replace contents in FA_MRM.csv and double-click to run the exe file.

**Figure 1. Partial contents of unzipped TAILOR-MS_MRM_generator folder→**

*Input file*

FA_MRM.csv must contain the following contents:

**Fatty_acid:** fatty acyl groups one intends to use for generating possible TG structures. “x” instead of “:” are used here so that Microsoft Excel does not automatically convert the input FAs to time.

**Reappearance:** indicates if the user allows an FA group to appear 2 or 3 times in a TG structure when generating MRM lists *eg* TG(18:1_18:1_18:1) and TG(8:0_18:1_18:1) will be allowed here but not TG(8:0_8:0_8:0).

**←Figure 2. An example for FA_MRM.csv**

*Output files*

TAILOR-MS MRM generator creates two files: **MRM_list.csv** and **TG_by_MRM.csv**.

Below are the contents of **MRM_list.csv:**

**Q1_Q3 Identity:** brutto level TGs and neutral loss FAs corresponding to Q1/Q3 pairs

**Q1:** Q1 m/z from LipidMaps

**Q3:** Q3 m/z from LipidMaps

**↑Figure 3. An example of MRM_list.csv**

TG_by_MRM.csv contains the following contents:

**No.:** numbers

**Brutto level TG:** brutto level TG

**TG structure:** structures with FA details

**↑Figure 4. An example of TG_by_MRM.csv**

**Identifier**

TAILOR-MS Identifier identifies and predicts TG structures with fatty acyl information but without positional information. It reads two input files: **“FA.csv”** and **“Input.csv”**. To use TAILOR-MS Identifier, simply replace the contents of these two csv files with your own data and preferred settings and double click **TAILOR-MS_Identifier.exe** to run the script.

First, unzip TAILOR-MS_Identifier.zip file and find the following files in the folder. Part of the folder should look like this:

**Figure 5. Partial contents of unzipped TAILOR-MS_Identifier folder→**

**↓Figure 6. An example of FA.csv file**

FA.csv must contain a single column with FA groups to be used for structural identification and prediction, similar to the first column of “FA_MRM.csv” file for MRM list generator.

Input.csv file has several columns and are explained below**:**

**Name:** anything the user wants to name their peaks. Must be English characters and numbers.

**TG:** brutto level TG.

**FA:** FA neutral losses.

**Peak:** used to assign peaks captured under the same Q1/Q3 which indicates chromatographic separation of structural isomers. Must be lower case English alphabets (a-z).

**↑Figure 7. An example of Input.csv file**

**RT_left:** left boarder of peak retention time. Must be 0 or positive values.

**RT_right:** right boarder of peak retention time. Must be positive values and greater than corresponding RT_left values.

**Intensity:** peak intensities, which can be areas, heights or concentrations. Must be positive values.

**Abundance_threshold(%):** a user-defined threshold. Any structures with a relative abundance (vs the peak with largest intensity of all peaks having the same TG brutto level) lower than this threshold will be removed. Must be 0 or positive values. All peaks with the same brutto level TG must have the same set value.

**RT_tolerance(%):** a user defined threshold. To confirm the existence of a TG structure, the constituting peaks of this structure must overlap. RT_tolerance enables the user to exclude TG structures of which the peaks do not overlap to the % above the set values. More detailed explanation can be found in TAILOR-MS paper. Must be positive values. All peaks with the same brutto level TG must have the same set value.

*Output file:*

Double click TAILOR-MS_Identifier.exe to start the run. Depending on the size of input data, this may take a few minutes.

**“Results.csv”** will be spawned in the same folder. Contents of this file are explained bellow.

**No.:** numbers

**Identification/Prediction:** indicate if the TG structure has been identified (having 3 FA groups in acquisition method and match the rules) or predicted (having only 2 FA groups)

**Brutto Level**: brutto level TG

**TG Structure:** TG structures with FA details

**Constructed Peaks:** correspond to “Peak” information from Input.csv. Capitalised letter(s) indicates the ID peak; # indicates missing peak from the acquisition method (predicted TG structures).

**ID Peak:** the FA peak with minimal intensity among the three FA groups that constitute a specific TG, which can be used to indicate the abundance of this particular TG structure.

**Name:** names from Input.csv.

**Retention Time:** retention time spans of ID peak.

**% Relative Abundance:** uncorrected relative abundances of ID peak.

**% Relative Abundance (corrected):** relative abundances corrected when ID peak reoccurs in a structure.

**Intensity:** uncorrected intensities of ID peak.

**Intensity (corrected):** intensities corrected when ID peak reoccurs in a structure.

**←Figure 8. An example of Results.csv**
